# War Opportunities

**Published:** 1918-11

**Authors:** 


### TEXAS MEDICAL JOURNAL

DR. F. E. DANIEL, Founder.

Established July, 1885

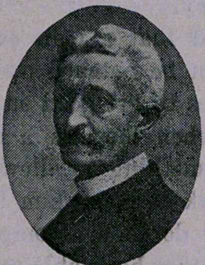

MRS. F. E. DANIEL, Publisher and Managing Editor.

VOL. XXXIV No. 5
AUSTIN, NOVEMBER, 1918.

PUBLISHED MONTHLY.
Subscription: $1.50

The publisher is not responsible for views of contributors.

ASSISTANT EDITORS:

DR. JOHN S. TURNER, Dallas, Texas. DR. JOE BECTON, Greenville, Texas.
DR. E. B. PARSONS, Palestine, Texas. DR. M. F. BLEDSOE, Port Arthur.
DR. A. P. HOWARD, Houston, Texas. DR. J. H. FLORENCE, Dallas, Texas.
DR. J. H. REUSS, Cuero, Texas.

*"All of good the past hath had,*

*Remains to make our own time glad."*—WHITTIER.

ARMY MEDICAL SEP 23 1947 LIBRARY

### EDITORIAL.

### War Opportunities.

Whether the war lasts much longer or not, it has brought and will continue to bring wonderful opportunities to the physicians and surgeons of the world. Of course those who have been directly engaged in war service in the cantonments of this country and at the front in Europe will secure the greatest benefits that can be gathered from large experience and practice, but these will return to their homes to carry back to other physicians the things they have learned.

Space is given this month in the Texas Medical Journal to a rather extended notice of the first meeting in this country of the Interallied Medical Congress. Perhaps the men from abroad who addressed that congress may not be any more able or any brighter than hundreds of our medical men on this side, but they had the advantage of being in the thickest of the fight where they have gained experience that could not otherwise be obtained in a life-time. Therefore they have seen and done things that are worth telling, and the telling will be of wonderful advantage to surgery. So, every surgeon who goes from America will come back rich in experiences of many kinds, and those experiences will be told again and again until the whole profession will profit thereby.

Surgeons have learned that a man can hardly be so badly wounded that he can not be patched up and made over into a new man, if only he lives to get good surgical attention. They are no longer content when they see a man shot or blown all to pieces to give him something to alleviate his suffering until he dies; they get to work to make him live, and in most cases they succeed. Not only do they accomplish this, but they restore him in such a way that he is able to do many of the things that a whole man can do.

It is not only in surgery that much has been learned; the profession has learned to cope with great epidemics as it would not otherwise have learned in a hundred years. Fifty years ago an epidemic like the Spanish influenza would have caused the death of more than half an army living under the conditions under which our men have been situated, and among the civilians ten would have died to where our losses have been one. Epidemics will no longer stagger the country. The government, acting through the physicians, will take serious epidemics in hand in future and see that they are immediately stamped out, for the government has come to appreciate the worth of its citizens. It may smack somewhat of paternalism—what if it does?—but hereafter the government will regard it as one of the greatest of governmental duties to see that the health of the people is safeguarded by every possible means at its command, and that regardless of what the cost may be. As a people we have learned that mere money is not to be counted against public health.

---

As usual when an epidemic strikes the country there are scores of people who claim to know just how to be immunized and how to cure it, and the papers are full of gratuitous advice from all sources. One claims that alcohol is a sure preventative and cure for Spanish influenza, and that the person who fills his system with drink can rest assured that he will not get the disease, and if he does get it, that he will not die. Every one knows that those who are addicted to drink have weakened constitutions, and that a weak constitution is in bad shape to resist disease. Another says that constant smokers do not have it. Perhaps he saves himself by the use of the word constant, for it might be hard for any kind of a disease germ to penetrate a tobacco-smoke barrage, since tobacco smoke sickens every thing that comes in contact with it, even men until they become smoke immune. Still another says that those who chew tobacco do not have it, the saliva being germ proof. Tobacco saliva will kill almost anything except the man who habituates himself to its use, but that does not prove that it will prevent disease. Dozens of other remedies equally as lacking in merit are offered, but people should be slow to rely on any of them.

---

Here is hoping that the war may soon be over—with the greatest of victories for the allies, of course—and that our physicians may be back at home once more, richer for their experiences, but as glad to get back as the people will be to see them.

---

Again the physicians of the country have demonstrated their unselfishness in attending upon the poor as faithfully as upon the rich in the epidemic of influenza through which the country has gone.

---

In the disposition to abbreviate words it does seem that the American people are determined to reach the limit. Just why the public—and physicians also—should take the perfectly good word ''influenza'' and make of it ''flu'' is inconceivable. Pretty soon they will be calling rheumatism ''rheu'' and neuralgia ''neu.''

---

''When the war is over,'' and it begins to look like that will be soon, the government, either national or state or city, or perhaps all, should get busy and see that the sanitary conditions in our communities and cities are made as they have been made around our army camps. The things that have been good for our soldiers will be just as good for the civilians, and it is just as necessary to look after the health of civilians as of the soldiers.

---

Now that this country has demonstrated its ability to raise money on a large scale to meet emergencies, why not put some of this governmental energy after the war into educating the people to having the best system of roads in the whole world. It would not be a bad idea to call upon the volunteers who have enlisted for the period of the war to extend their enlistment until they can construct a complete system of good roads under national or state direction. The people would not hesitate to buy bonds authorized for such purposes.

